# Identification of Efficacy-Associated Markers to Discriminate *Flos Chrysanthemum* and *Flos Chrysanthemi Indici* Based on Fingerprint–Activity Relationship Modeling: A Combined Evaluation over Chemical Consistence and Quality Consistence

**DOI:** 10.3390/molecules28176254

**Published:** 2023-08-25

**Authors:** Feng Liu, Yuanrong Zheng, Huijie Hong, Lianliang Liu, Xiaojia Chen, Qiang Xia

**Affiliations:** 1 Department of Horticultural Technology, Ningbo City College of Vocational Technology, Ningbo 315100, China; 2 State Key Laboratory of Dairy Biotechnology, Shanghai Engineering Research Center of Dairy Biotechnology, Dairy Research Institute, Bright Dairy & Food Co., Ltd., Shanghai 200436, China; 3 State Key Laboratory of Quality Research in Chinese Medicine, Institute of Chinese Medical Sciences, University of Macau, Macau 999078, China; 4 College of Food and Pharmaceutical Sciences, Key Laboratory of Animal Protein Food Processing Technology of Zhejiang Province, Ningbo University, Ningbo 315832, China

**Keywords:** *Chrysanthemum*, antioxidant, cellular antioxidant activity, α-glucosidase inhibitory activity, lipase-inhibitory activity, fingerprint–activity relationship

## Abstract

Monitoring the quality consistency of traditional Chinese medicines, or herbal medicines (HMs), is the basis of assuring the efficacy and safety of HMs during clinical applications. The purpose of this work was to characterize the difference in hydrophilic antioxidants and related bioactivities between *Flos Chrysanthemum* (JH) and its wild relatives (*Chrysanthemum indicum* L.; YJH) based on the establishment of fingerprint–efficacy relationship modeling. The concentrations of the total phenolics and flavonoids of JH samples were shown to be generally higher than those of YJH, but the concentration distribution ranges of YJH were significantly greater compared to JH samples, possibly related to environmental stress factors leading to the concentration fluctuations of phytochemicals during the growth and flowering of *Chrysanthemum* cultivars. Correspondingly, the total antioxidant capabilities of JH were greatly higher than those of YJH samples, as revealed by chemical assays, including DPPH and ABTS radical scavenging activities and FRAP assays. In addition, cellular-based antioxidant activities confirmed the results of chemical assays, suggesting that the differences in antioxidant activities among the different types of *Chrysanthemums* were obvious. The extracts from YJH and JH samples showed significant α-glucosidase inhibitory activity and lipase-inhibitory activity, implying the modulatory effects on lipid and glucose metabolisms, which were also confirmed by an untargeted cell-based metabolomics approach. The selected common peaks by similarity analysis contributed to the discrimination of YJH and JH samples, and the modeling of the fingerprint–bioactivity relationship identified neochlorogenic acid, isochlorogenic acid A, and linarin as efficacy-associated chemical markers. These results have demonstrated that integrating HPLC fingerprints and the analysis of similarity indexes coupled with antioxidant activities and enzyme-inhibitory activities provides a rapid and effective approach to monitoring the quality consistency of YJH/JH samples.

## 1. Introduction

*Flos Chrysanthemum* (Juhua, JH) is the Latin term for *Chrysanthemum* flower, referring to cultivated chrysanthemum flowering heads used as food and traditional Chinese medicines (TCMs). Wild chrysanthemums (Yejuhua, YJH), on the other hand, are the naturally occurring, uncultivated species of chrysanthemums found in the wild [1]. Both types of *Chrysanthemums* can used as inherent components in different TCM formulas, attributed to their multiple bioactivities such as antibacterial, anti-inflammatory, anticancer, hepatoprotective, antiallergy, antioxidant, immunomodulatory, neuroprotective, and antimicrobial activities [2,3,4]. *Chrysanthemum* belongs to the *Asteraceae* genera, as one of the biggest plant families, showing significant chemical variability accompanied by different origins, cultivars, and environment conditions [2]. Although there are many similarities shared in appearance, bioactivities (e.g., heat-cleaning, brightening eyes, toxin expelling), and bioactive components related to pharmaceutical properties as identified in recent years for JH and YJH [5,6,7], their comparative studies aimed at achieving quality consistency monitoring have been scarcely reported hitherto.

As a typical metabolic disease, diabetes mellitus (DM) is related to either impaired insulin secretion or insulin resistance, with the majority of DM patients being type II diabetes associated with pancreatic β-cell abnormalities and insulin resistance [8,9]. To aid in controlling blood sugar levels for type II diabetes, the use of an α-glucosidase inhibitor is a general strategy to suppress carbohydrate absorption and prevent blood sugar elevation by competitively inhibiting the enzyme α-glucosidase responsible for converting complex dietary carbohydrates into absorbable monosaccharides [10]. Obesity is often interacted with by DM. So, when examining the potential bioactivities of plant extracts, α-glucosidase and lipase-inhibitory activities are in general characterized simultaneously in terms of their capability to inhibit pancreatic lipase for reduced lipid absorption via suppressing the conversion of triglycerides to free fatty acids [11].

Due to the extreme complexity of phytochemicals occurring in TCM materials and the integrated role of multicomponent and multitargets during clinical practices, only identifying and quantifying several limited bioactive components for chemical profiling and pharmacological characterization is not adequate or effective to evaluate the quality consistency of TCMs [12]. Therefore, fingerprint–efficacy relationship modeling has been widely used for the quality evaluation of TCM materials. The general strategy to discover quality-associated markers by fingerprint–efficacy relationship modeling involves chemical profiling, bioactivity characterization, and fingerprinting–efficacy model establishment, as well as discovery–verification of markers [12,13]. To establish the model, different types of multivariate analysis, such as partial least-squares regression (PLSR) analysis and artificial neural network (ANN), are employed to discover efficacy-associated markers [14,15].

This work aimed at achieving accurate quality discrimination between different types of *Chrysanthemum* cultivars, relying on the methodology of fingerprint–activity relationship modeling to search for potential chemical markers. Initially, the antioxidant activities and glucosidase and lipase-inhibitory activities were analyzed. Then, the chromatographic fingerprints were obtained by high-performance liquid chromatography coupled with diode-array detection (HPLC-DAD) with subsequent identification of common featured chromatographic peaks by measuring similarity indexes. Finally, the potential correlation model between the common characteristic peaks distinguished within the fingerprints and the bioactivities expressed as antioxidant capabilities and enzyme-inhibitory activities was established by PLSR and BP-ANN.

## 2. Results and Discussion

### 2.1. Chromatographic Fingerprints and the Analysis of Similarity

**Figure 1:**
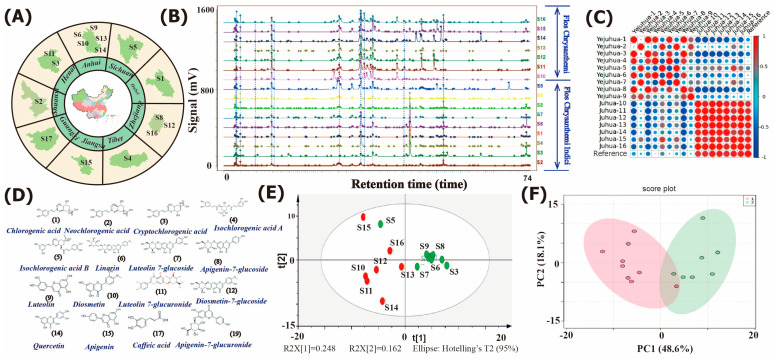
Geographical information for the collected samples (**A**); HPLC-DAD chromatographic profiles acquired at 280 nm for *Chrysanthemum* (**B**); similarity index (**C**); the chemical structures of the isolated bioactive compounds (**D**); the PCA models established by HPLC fingerprint profile data (**E**) and the identified (**F**).

The chromatographic fingerprints of 16 *Chrysanthemum* samples with different origins were obtained, where 110 and 65 chromatographic peaks were identified for YJH and JH samples, respectively (Figure 1A,B), suggesting that the metabolite composition of YJH was possibly more complex than that of JH samples. On the other hand, the relative peak abundance of the identified compounds found in YJH was significantly lower than that in JH samples. This observation may support the claim that environmental stress factors contribute to the biosynthesis of secondary metabolites under natural field conditions and significant fluctuations in their contents thus explaining the complex composition and rich distribution of secondary metabolites in YJH samples, simultaneously coinciding well with multiple pharmacological activities of YJH including antibacterial, anti-inflammatory, anticancer, hepatoprotective, antiallergy, antioxidant, immunomodulatory, neuroprotective, and antimicrobial activities [16,17,18,19]. As a wild resource, YJH is rich and widely distributed in wild places such as mountain slopes, grasslands and shrubbery, and roadsides [18]. Comparatively, JH samples (*Flos Chrysanthemum*) are generally from cultivated farms, with abiotic stresses scarcely encountered during growth, and therefore, completely differential environmental conditions resulted in significant variations in the distribution of bioactive phytometabolites and chemodiversity.

To initially compare the differences between YJH and JH samples, the HPLC chromatography of all the *Chrysanthemum* samples was subjected to similarity analysis (Figure 1B), with 16 compounds identified as the common peaks, including chlorogenic acid, neochlorogenic acid, cryptochlorogenic acid, isochlorogenic acid A, isochlorogenic acid B, linarin, luteolin 7-glucoside, apigenin-7-glucoside, luteolin, diosmetin, luteolin 7-glucuronide, diosmetin-7-glucoside, quercetin, apigenin, caffeic acid, and apigenin-7-glucuronide (Figure 1). PCA score plots of the 16 chrysanthemum samples were successfully separated according to all the peaks detected from HPLC chromatographic profiles, but according to the major common peaks, the samples could be separated into sub-groups of YJH and JH on the two-dimensional plane of the score (Figure 1E,F).

### 2.2. In Vitro Antioxidant Activity Characterization Based on Chemical Methodologies

**Figure 2:**
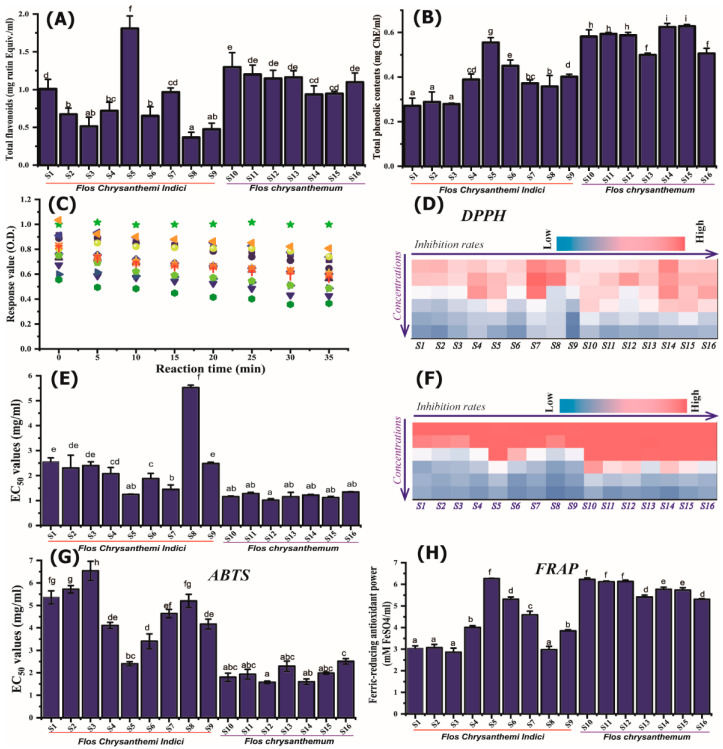
Differential distribution of the contents of total phenolics (**A**) and total flavonoids (**B**) in *Flos Chrysanthemi Indici* and *Flos Chrysanthemi;* (**C**) the free radical scavenging kinetics by bioactive components of different samples; (**D**) the relationship between DPPH free radical scavenging capability and the samples concentration ranging between 0.625 and 20 mg/mL; (**E**) EC_50_ values for DPPH; (**F**) the relationship between ABTS free radical scavenging capability and the samples concentration; (**G**) EC_50_ values for ABTS; (**H**) FRAP values of the extract from different samples. Different lower-case letters in the figure denote significance with *p* < 0.05.

Based on the relationship between the free radical scavenging rate of ABTS and DPPH and the concentration of the extract (Figure 2B,D), the median effective concentrations (EC_50_) values of the two were calculated (Figure 2C,E). The results showed that there were significant differences among different samples, especially the different varieties of wild chrysanthemum. Among the selected YJH samples, the EC_50_ values of S1–S4, and S8 were significantly different (S1–S9), among which the EC_50_ values of the S1–S4 and S8 samples were higher, and the S5 sample was the lowest, indicating that S1–S4 and S8 had weak antioxidant activities, and S5 presented higher antioxidant activities; while different varieties The free radical scavenging rate among chrysanthemums is relatively uniform, and the difference is not significant (such as among the S11–S16 samples). Overall, the free radical scavenging rate EC_50_ value of wild chrysanthemum is higher than that of chrysanthemum, and the results of the two free radical scavenging rates are consistent, indicating that the antioxidant capacity of chrysanthemum is stronger than that of wild chrysanthemum, which corresponds to the distribution of flavonoids.

Free radical scavenging capability measurement suggested a linear concentration and reaction time-dependent relationship. The free radical scavenging kinetics by bioactive components were examined as kinetic behavior is affected by chemical composition, sample sources, and concentration: a fast decay in absorbance in the first 5 min; all samples showed a steady state after 30–35 min. Based on the EC_50_ values calculated, Yejuhua presented significantly higher EC_50_ values than Juhau extract, which exhibited no significant difference among the different sources, suggesting the selected JH demonstrated stronger antioxidant activities than YJH.

The results of total antioxidant capacity characterized by FRAP (Figure 2F) are consistent with the results of antioxidant activity characterized by ABTS and DPPH free radical activity, consistently indicating that the antioxidant capacity of the S1, S2, S3, and S8 samples was significantly weaker than other samples while S5 showed the highest values. The chrysanthemum samples (JH) showed comparable antioxidant capacity to other chrysanthemum samples; the FRAP values of chrysanthemums from different sources were less varied than wild chrysanthemum samples, and the values of the S13 and S16 samples were the lowest, which were completely consistent with the results of EC_50_ values expressed by ABTS assays.

In addition, the distribution of the antioxidant activity of different samples characterized by different methods showed a close relationship with the results of the total flavonoid contents and total phenolics. For example, the S5 sample with higher activity shows the highest content of total flavonoids, corresponding with the relatively high EC_50_ values characterized by DPPH for the S5 sample. Similarly, S8 demonstrated the lowest total flavonoid contents, matched well with the highest EC_50_ values as expressed by DPPH assays. The difference in antioxidant activity of chrysanthemum samples is weaker than that of wild chrysanthemum, which can be attributed to the difference in flavonoid content distribution.

### 2.3. Cellular-Based Antioxidant Activity Characterization

**Figure 3:**
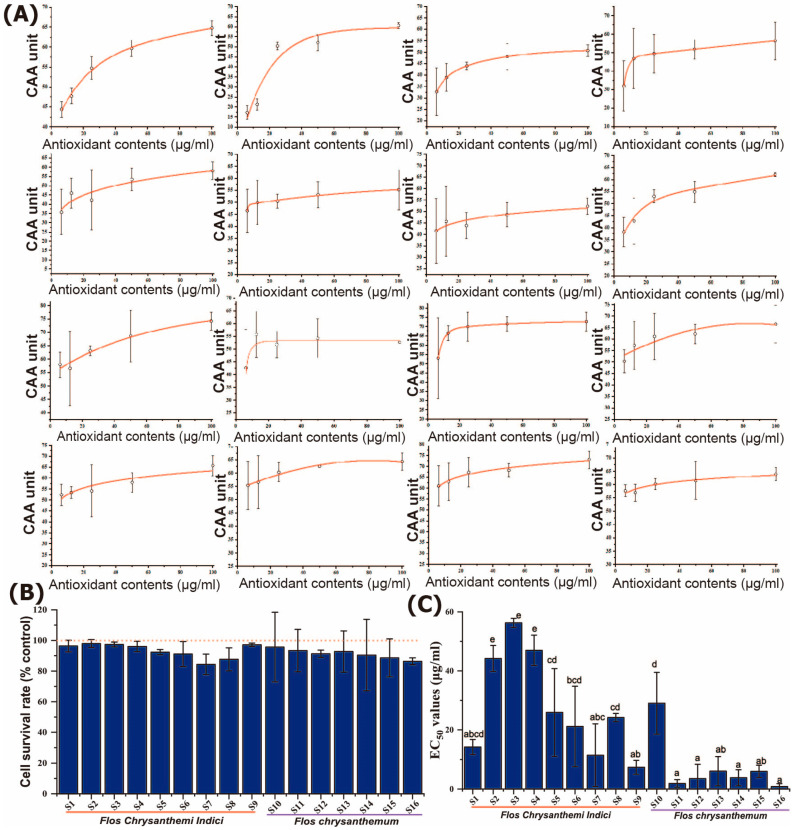
Antioxidative effects of different sample extract on 2,2-azobis (2-methylpropionamidine) dihydrochloride (ABAP)-damaged HepG2 cells: relation of CAA units with antioxidant contents (**A**); cck-8 cytotoxicity assay (**B**); EC_50_ values based on CAA methodology (**C**). Different lower-case letters in the figure denote significance with *p* < 0.05.

According to the dose–response curve and intermediate effect principle of the CCA value under different concentration gradients of the major common peaks (Figure 3A), the EC_50_ values of the extraction solution were obtained. The CCK-8 assay was used to detect the cell viability of each group after adding the extraction solution. The concentration of the extraction solution was determined based on the cell viability under different concentration gradients. Under the selected concentrations, the cell survival rates were not significantly (*p* ≥ 0.05) influenced, thus assuring the independence of the CAA values on the potential toxicity of the YJH/JH extracts (Figure 3B). The EC_50_ values of YJH samples were shown to be significantly higher than those of JH samples (Figure 3C), indicating that the antioxidant activities of JH were higher than those of YJH, in line with the observation results obtained from chemical-based evaluation approaches, including ABTS, FRAP, and DPPH. Although universal and accurate approaches to antioxidant activities evaluation remain limited, it has been well recognized by substantial research that using cell-based approaches has its advantages over in vitro chemical evaluation approaches [20,21]. Similarly, CAA analysis was used to characterize the antioxidant activities of the ethanol and water extracts of *Lactobacillus plantarum* Y16 fermented soymilk, coinciding well with the results of the radical scavenging ability of hydroxyl and DPPH radicals [22]. The consistence between multiple in vitro chemical antioxidant assays and CAA assays further confirms that JH possesses greatly higher antioxidant activities than those of YJH.

### 2.4. Glucosidase Inhibitory Activity and Lipase-Inhibitory Activity

**Figure 4:**
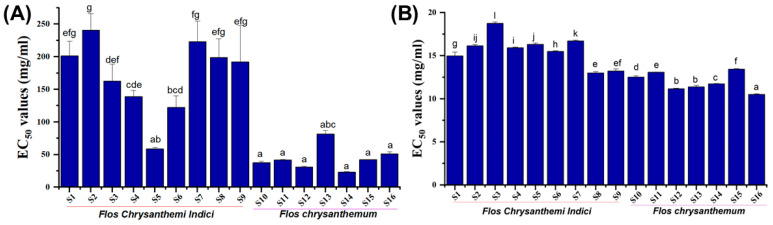
Glucosidase inhibitory activity (**A**) and lipase-inhibitory activity (**B**) of the extracts. Different lower-case letters in the figure denote significance with *p* < 0.05.

The use of plant extracts is generally considered as an efficient methodology to manipulate the glucose contents of postprandial plasma through inhibiting the enzymes such as α-amylase and α-glucosidase [9,23]. The results of enzyme-inhibitory activities are presented in Figure 4. Regarding glucosidase inhibitory activity, the fluctuations in EC_50_ values of YJH samples were more prominent than those of JH samples. Except for the S5 sample, the inhibitory rates of wild chrysanthemum (YJH) extracts on glycosidase activities were significantly lower (*p* < 0.05) than those of chrysanthemum. Similarly, the EC_50_ values of YJH samples to inhibit lipase activities were generally higher than those of JH samples, suggesting that JH tended to possess more abundant bioactive components than YJH for inhibiting the enzymes. On the other hand, the difference between different wild chrysanthemum varieties (YJH) was much larger than those of chrysanthemum (JH), and there was no significant difference in the glycosidase-inhibitory activities of different chrysanthemums. The above-mentioned results were consistent with the results obtained from the total flavonoids, as well as the distribution patterns of total phenolics. However, the varied distribution of EC_50_ values in glycosidase-inhibitory activities among different samples may suggest that the inhibition of glycosidase could be more dependent on the distribution of total flavonoids compared with total phenolics, as previously observed in α-glucosidase and lipase-inhibitory activity of the phenolic substances in black legumes from different genera [24].

### 2.5. Identification of Potential Bioactive Markers by PLSR and ANN

Chemometric methods such as PLSR and ANN are employed to search for efficacy-associated bioactive components or quality control markers by establishing a fingerprint–efficacy model [12,25]. The association between the bioactive attributes, including antioxidant capability and α-glucosidase-/lipase-inhibitory activities, and the characteristic peaks was modeled by PLSR and BP-ANN.

**Figure 5:**
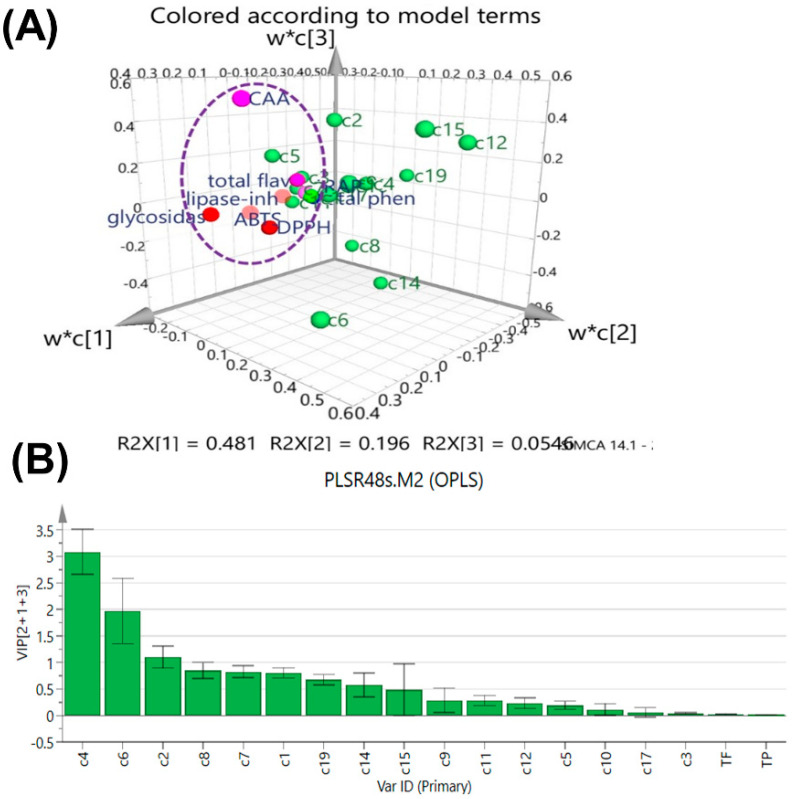
Loadings scatter plot (**A**) and variable influence on projection (VIP) (**B**) for the established partial least-squares regression (PLSR) model: R^2^X(cum) = 0.895, R^2^Y(cum) = 0.829, Q^2^ = 0.8.

A PLSR model to elucidate the contribution of phytocomponents to bioactivities was established by correlating antioxidant and enzyme-inhibitory activities (Y-matrix) to the chromatographic data of the sample matrix (16 × 16). The 3D plot of the PLSR loading scatter is shown in Figure 5A, where 89.5% of the variance in X-variables and 82.9% of the variance in Y-variables are explained, with three latent variables explaining 48.1%, 19.6%, and 5.46% of the variations for the first, second, and third latent variables, respectively. Y-variables were situated around the selected X-variables. Particularly, the antioxidant and enzyme-inhibitory activities were close to most of the chemical compounds and distributed together in the same cluster, suggesting that Y-variables were positively related to X-variables. Therefore, the values of variable importance for the projection (VIP) were used to screen the X-variables which were quantitatively and statistically important to the Y-variables [26]. In general, VIP values higher than 1.0 are considered important [27,28], and according to this basis, the peaks **C2**, **C4**, and **C6** were important loading contributions thus corresponding to high correlation with the bioactivities of YJH and JH samples. Due to the possible presence of synergistic or antagonism effects among different variables, a nonlinear ANN model was established to further reveal the fingerprint–efficacy relationship in virtue of its capability to achieve pattern recognition, self-adaptation, and parallel processing of fingerprint data, related to the information processing of three layers, including the input layer, the hidden layer, and the output layer [14]. The established BP-ANN model demonstrated good performance revealed by the MSE values of training, validation, and testing: 0.0001, 0.0090, and 0.0352, respectively. The contribution ratios of the detected common peaks further confirmed the results of the PLSR model. Therefore, based on the results obtained from PLSR and BP-ANN, neochlorogenic acid, isochlorogenic acid A, and linarin were chosen as antioxidant activities-related markers for JH and YJH samples.

### 2.6. Effects of the Identified Bioactive Markers on the Cell Metabolome

**Figure 6:**
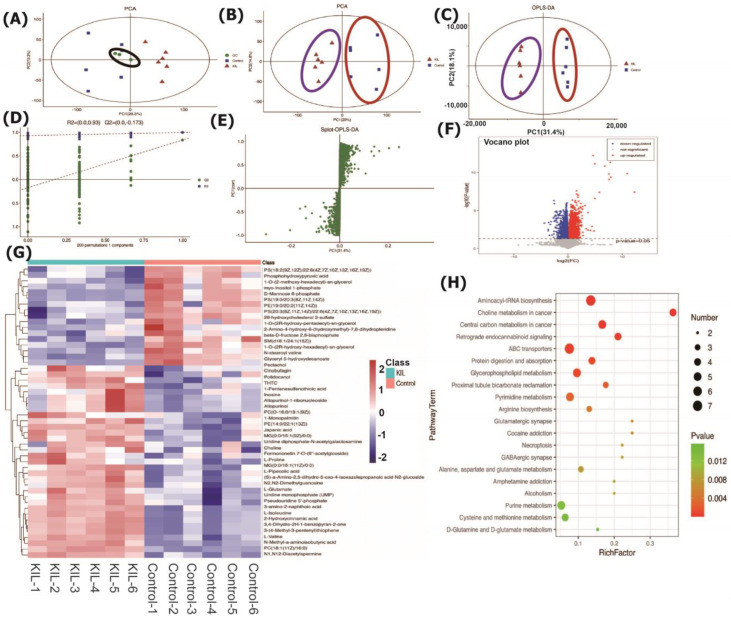
Metabolome changes in the HepG2 cells of bioactive molecules: (**A**) the clustering of QC samples in two-dimensional PCA; (**B**) PCA analysis based on the cell metabolome; (**C**) the orthogonal partial least-squares discriminant analysis (OPLS-DA); (**D**) random permutation test with 200 permutations for the OPLS-DA model; (**E**) S-plot; (**F**) volcano map; (**G**) the clustered heat map; (**H**) KEGG analysis.

Untargeted profiling of the metabolites of in vitro cultured HepG2 cells was performed to further confirm the metabolic changes and potential biomarkers associated with glycometabolism and lipid metabolism as influenced by the identified major bioactive components, including neochlorogenic acid, isochlorogenic acid A, and linarin. PCA is an unsupervised data analysis method that utilizes an orthogonal transformation to convert the original random vectors with correlated components into new random vectors with uncorrelated components [27,29,30,31]. This transformation aims to retain as much of the original variable information as possible, thereby achieving dimensionality reduction. Simultaneously, system stability was evaluated. As shown in Figure 6, the PCA model graph obtained through sevenfold cross-validation demonstrated that the QC samples were closely clustered together (Figure 6A,B), indicating good instrument detection stability during the experimental process.

To process the extracted data, ion peaks with missing values (0 values) exceeding 50% within each group were removed. Those 0 values with half of the minimum values were replaced. Then, based on the qualitative results of compound identification, a score (Score) and filter of the compounds were assigned, with a score of 36 (out of a maximum of 60 points) as the screening criterion. Compounds scoring below 36 are considered to have inaccurate qualitative results and should be removed. Finally, the positive and negative ion data were merged into a single-data matrix table, which included all the information extracted from the original data that could be used for analysis.

A combined approach of multivariate and univariate analyses was employed to identify differentially expressed metabolites between groups (Figure 6C), with permutation testing to confirm the quality of the established model (Figure 6D). In the orthogonal projection to latent structure-discriminant analysis (OPLS-DA), the variable important in projection (VIP) values were used to assess the impact strength and explanatory power of each metabolite’s expression pattern on the classification and discrimination of samples between groups. Significant metabolites with biological relevance were identified by exploring VIP values and further validated for significance using a *t*-test to determine inter-group differences (Figure 6F). The generated S-plot could be used to further validate the potential metabolites of great interest (Figure 6E). The clear separation between HepG samples without extract addition and the cells with additions suggested significantly differential effects on the HepG2 cell metabolome, according to the significantly altered contents of intracellular metabolites after exposing the cells to the YJH/JH extracts. In addition, the complete separation of PCA contributed to the increased reliability of the conclusions obtained from OPLS-DA [27,31,32]. In order to visualize the relationships between samples and the expression differences of metabolites across different samples, we performed hierarchical clustering on all significantly differentially expressed metabolites. The results are shown in the diagram of Figure 6G. The horizontal axis represents sample names, while the vertical axis represents differentially expressed metabolites. The color gradient from blue to red indicates the abundance of metabolite expression, with red indicating higher expression levels of differentially expressed metabolites. A total of 69 lipids and lipid-like molecules of 184 differential metabolites were identified (Figure 6G). Among the major pathways affected by the extracts, glycerophospholipid and central carbon metabolism were included (Figure 6H). So far, to our best knowledge, there is no literature examining the effects of extracts from *Flos Chrysanthemum* and wild *Chrysanthemum* (*Chrysanthemum indicum* L.) on the cell metabolism of HepG2. Previously, it has been demonstrated that a cell-based metabonomics approach is effective in investigating the potential metabolic effects of phytometabolites on the modifications of differential intracellular metabolites and the disrupted metabolic pathways, as exemplified in the case of chrysophanol-8-*O*-β-d-glucoside [33] and drug-induced causes of hepatotoxicity during preclinical testing using liver cell models [34]. The cell-based metabonomics corresponded well with the results of α-glucosidase- and lipase-inhibitory activities, exhibiting the regulatory effects of the *Chrysanthemum* extracts on lipid and glucose metabolism.

## 3. Materials and Methods

### 3.1. Materials, Sample Preparation, and Chemicals

The powder of the samples (0.50 g) was accurately weighed through a 10-mesh sieve and added by 25 mL of 70% methanol in a stoppered conical flask, followed by an ultrasonic extraction step (SCIENTZ SB-300 DTY, Ningbo Scientz Biotechnology, Ningbo, China) at 300 W and 45 kHz for 40 min. After the extraction was finished, the methanol (70%, *v*/*v*) was supplemented to achieve the initial volume, with the filtrate to obtain the final product. All chemicals and solvents were of analytical or HPLC grade. Water, methanol, acetonitrile, and formic acid were obtained from Thermo Fisher Scientific (Waltham, MA, USA). L-2-chlorophenylalanine was from Shanghai Hengchuang Bio-Technology Co., Ltd. (Shanghai, China). Chloroform was from Titan Chemical Reagent Co., Ltd. (Shanghai, China). The standard compounds, including chlorogenic acid (**C1**), neochlorogenic acid (**C2**), cryptochlorogenic acid (**C3**), isochlorogenic acid A (**C4**), isochlorogenic acid B (**C5**), linarin (**C6**), luteolin 7-glucoside (**C7**), apigenin-7-glucoside (**C8**), luteolin (**C9**), diosmetin (**C10**), luteolin 7-glucuronide (**C11**), diosmetin-7-glucoside (**C12**), quercetin (**C14**), apigenin (**C15**), caffeic acid (**C17**), and apigenin-7-glucuronide (**C19**), were obtained from Chengdu Purify and Pufei De Biotech Co., Ltd. (Chengdu, China). The free radicals used for antioxidant activity assays, 2,2-diphenyl-1-picrylhydrazyl (DPPH) and 2,2′-azinobis(3-ethylbenzothiazoline 6-sulfonate; ABTS), were purchased from Aladdin Bio-Chem Technology Co., Ltd. (Shanghai, China). A total of 16 samples, involving *Flos Chrysanthemum* and wild *Chrysanthemum*, were collected and the sample information is shown in Table S1.

### 3.2. Chromatographic Conditions

The bioactive components of the extracts from *Flos Chrysanthemum* and wild *Chrysanthemum* samples were isolated using an Agilent 1200 HPLC equipped with a diode-array detector, C_18_ column (4.6 mm × 250 mm, 5 μm), autosampler, column compartment, and Agilent ChemStation for data analysis. The mobile phase included the combination of B (0.1% formic acid in acetonitrile) and A (0.1% formic acid in ultra-purified water) using a linear gradient elution as follows: 0–40 min (90–74% A); 40–70 min (74–35% A); 70–71 min (30–0% A); and 71–75 min (0% A). The flow rate was set at 0.8 mL/min. The column and sample temperatures were 25 °C. The injection volume was 10 μL. More than 8000 theoretical plates were obtained for 3,5-di-*O*-caffeoylquinic acid. The HPLC-DAD at 280, 320, 350, and 520 nm was performed for real-time monitoring of the intensities of chromatographic peaks. The detected major peaks were qualitatively and quantitatively identified by comparing them with the standard compounds of which the separate stock solutions (10–100 μg/mL) were prepared.

### 3.3. Determination of Antioxidant Components

Total flavonoids of the extracts from *Flos Chrysanthemum* and wild *Chrysanthemum* samples were measured according to the previously well-established method. Rutin standard curve (*y* = 0.7604*x* − 0.2294, *R*^2^ = 0.9889). To determine the contents, a total of 100 μL of diluted sample was added to 2 mL of 2% Na_2_CO_3_ aqueous solution. After 2 min, 100 μL of 50% Folin–Ciocalteau reagent was added. The final mixture was shaken and then incubated at room temperature for 30 min in the dark at room temperature. The absorbance of all samples was measured at 750 nm using a microplate reader, and the results are expressed in mg chlorogenic acid equivalents per gram extract (mg ChE/mL extract) with eight replicates of measurements. The standard curve of the equivalent compound for calibration purposes was obtained (*y* = 1.323*x* − 0.0869, *R*^2^ = 0.9923).

### 3.4. Chemical Evaluation of In Vitro Antioxidant Activities

Total antioxidant capacities of different samples were assessed by determining DPPH and ABTS radicals scavenging activities and ferric-reducing antioxidant power (FRAP) assays, according to the previously reported methodologies [28]. Fifty microliters of oil sample at various concentrations (10–200 μg/mL) was added to a methanol solution of DPPH. A 50 μL volume of the sample was reacted with 150 μL of 0.2 mM DPPH solution. Absorbance measurements were taken 6 min after the reaction at 517 nm using a Flex Station III Multi-Mode Microplate Reader (Molecular Devices, San Jose, CA, USA). The radical scavenging activity was calculated by the DPPH inhibition percentage as follows: %DPPH radical scavenging = 100(1 − B/A), where *A* and *B* are the blank and oil sample absorbance.

The ABTS was measured by pre-formed radical monocation. The mixtures, along with 7.4 mM ABTS solution and 2.6 mM potassium persulfate, were incubated at room temperature in the dark for 24 h. The ABTS solution was diluted with phosphate-buffered saline (pH 7.4) to achieve an absorbance of 0.7 ± 0.02 at 734 nm. Each sample was suspended in distilled water, and 40 μL of the sample was reacted with 160 μL of the ABTS solution. Absorbance was taken 6 min after the reaction at 734 nm.

FRAP assay was performed using a commercial kit (A015-3-1, Nanjing Jiancheng Bioengineering Institute, Nanjing, China). 10 μL of each sample solution was mixed with 180 μL of newly prepared FRAP reagent. After incubation at 37 °C for 5 min, the absorbance of every reaction mixture was recorded at 593 nm.

### 3.5. Cellular-Based Antioxidant Capacity Evaluation

Following the method of Wolfe and Liu [35], a cellular antioxidant activity (CAA) assay was performed using HepG2 cells cultured in minimum Eagle’s medium, including the blank group, the control group, and the experimental group supplemented with the extracts containing bioactive components. Three parallel wells were set up in each group. The cell suspension was inoculated at 100 μL per well (5 × 10^3^ cells per well) and cultured for 24 h. After the incubation, the growth medium was discarded, and the cells were washed to eliminate dead and unattached cells. Then, 100 μL of extracts containing different concentrations of the bioactive ingredients (1.25, 2.50, 5.00, 10.00, and 20.00 μmol·L^−1^) and 25 μmol 2′,7′-dichlorofluorescein diacetate (DCFH-DA) solutions to the experimental group. For the blank and control group, 100 μL of solutions containing only 25 μ mol/L DCFH -DA was added. After an incubation process at 37 °C and 5% CO_2_ for 1 h, cells were washed to remove the spare DCFH-DA. The control group and the experimental group were treated by adding 100 μL of HBSS solution containing 600 μmol/L 2,2-azobis (2-amidinopropane) dihydrochloride (AAPH) as a free radical generator [36], and the blank group was added with 100 μL of HBSS solution. The real-time fluorescence of the wells was recorded using a fluorescent microplate reader at the excitation wavelength of 485 nm and emission wavelength of 528 nm (Tecan, Infinite™ M200 PRO, Männedorf, Switzerland) with the fluorescence value measured every 5 min for 1 h. The CAA unit for quantitatively assessing cellular antioxidants was defined with the following equation:

CAA unit = 1 − (∫ SA/∫ CA)(1)

where ∫ SA represents the cumulative area of the sample curve, and ∫ CA indicates the cumulative area of the control curve.

### 3.6. Measurement of α-Glucosidase Inhibitory Activities

α-Glucosidase inhibition activities of the different sample extracts were characterized based on the procedures described by Uraipong and Zhao [37]. Aliquots of 100 μL of the extracted solutions were mixed evenly with 250 μL α-glucosidase solution (2 IU/mL), followed by an incubation in a constant-temperature water bath at 37 °C for 10 min, then 250 μL p-nitrophenyliu-D-galactopyranoside (PNPG) was added. The reaction was initiated with glucopyranoside (pNPG) (5.0 mM) solution, 250 μL of Na_2_CO_3_ (1 M) was added immediately to terminate the reaction after a 15 min reaction process at 37 °C, and the absorbance was detected at 405 nm with the following equation to calculate the α-glucosidase inhibitory activities (GIAs)

GIA (%) = [1 − (As − An)/Ac] × 100(2)

where A_S_, A_n_, and Ac represent the absorbance value of the solutions added with the enzyme, the extracts, and pNPG; the solutions added with the extracts and pNPG; and the solutions of the enzyme and pNPG, respectively.

### 3.7. Measurement of Lipase-Inhibitory Activities

The lipase inhibitory activities of different extracts were compared following the protocol as described by Ahmed [38] with few modifications. *p*-Nitrophenyl palmitate (*p*-NPP) was used as the substrate, which was hydrolyzed to form *p*-nitrophenol, a colored component that can be monitored under 405 nm. The extract solutions were mixed with pancrelipase solution (1000 IU/mL) and incubated at 37 °C for 15 min, followed by the addition of 200 μL of *p*-NPP solution (2.0 mM/L). After the reaction was completed, the solutions were placed at 100 °C for 5 min to terminate the reaction. The terminated reaction solution was centrifuged (5000× *g*, 5 min) to obtain the supernatant, and the absorbance of the solution was recorded. Orlistat was used as the positive control, and each test was performed in three duplicates. The following formula was used to calculate the lipase-inhibitory activities:

Inhibition rate (%) = [1 − (As − An)/Ac] × 100(3)

where As, An, and Ac represent the absorbance of the solutions containing lipase and extracts, the extracts without lipase, and the extracts with lipase, respectively.

### 3.8. Modeling of Fingerprint–Activity Relationship

A PLSR model was built to explore the fingerprint–activity relationship and the discrimination between *Flos Chrysanthemum* and wild *Chrysanthemum*. PLSR is a statistical technique used for regression modeling, especially when dealing with high-dimensional data sets with a large number of predictor variables (features) and potential multicollinearity among these variables, through obtaining linear combinations of the original predictor variables (X) that best explain the variation in the response variable (Y). In this study, the X-variables were the identified common peaks, while the Y-variables included antioxidant activities expressed by chemical approaches, CCA values, and enzyme-inhibitory activities.

The BP-ANN algorithm was used to model nonlinear relationships between inputs of HPLC fingerprint and outputs of bioactivities. The BP-ANN model was established by coding in Python software programming language, where the Levenberg–Marquardt back-propagation algorithm was used for network training:

*X*_*k*+1_ = *X_k_* − (*J^T^J* + *μI*)^−1^*J^T^e* (4)

where *J* represents the Jacobian matrix, *I* means the identity matrix, and *e* indicates a vector of network errors.

### 3.9. Metabolomic Analysis

Metabolite profiling analysis of HepG2 cells was performed to validate the potential metabolic pathways, as influenced by the identified chemical markers. The methanol solution (80%, *v*/*v*) was added to each sample and transferred to a 4 mL glass vial, followed by the addition of chloroform and sample dispersion. The cells were subjected to an ultrasonic homogenizer for 6 min at 500 W. All of the mixtures of each sample were transferred to 1.5 mL Eppendorf tubes, and L-2-chlorophenylalanine (0.3 mg/mL) dissolved in methanol was used as internal standard, then extracted by ultrasonication for 20 min in an ice-water bath. The extract was centrifuged at 4 °C (13,000 rpm) for 10 min. The supernatant in a glass vial was dried in a freeze-concentration centrifugal dryer. The mixture of methanol and water (1/4, vol/vol) was added to each sample, samples vortexed for 30 s, extracted by ultrasonic for 3 min in an ice-water bath, then placed at −20 °C for 2 h. Samples were centrifuged at 4 °C (13,000 rpm) for 10 min. The supernatants from each tube were collected using crystal syringes, filtered through 0.22 μm microfilters, and transferred to LC vials. The vials were stored at −80 °C until LC-MS analysis. QC samples were prepared by mixing an aliquot of all the samples to form a pooled sample.

A Nexera UPLC system (Shimadzu Corporation, Kyoto, Japan) coupled with a Q-exactive quadrupole-orbitrap mass spectrometer equipped with heated electrospray ionization source (Thermo Fisher Scientific, Waltham, MA, USA) was used to analyze the metabolic profiling in both ESI-positive and ESI-negative ion modes. An ACQUITYUPLC HSS T3 column (1.8 μm, 2.1 × 100 mm) was employed in both positive and negative modes. The binary gradient elution system consisted of (A) water (containing 0.1% formic acid, *v*/*v*) and (B) acetonitrile (containing 0.1% formic acid, *v*/*v*) and separation was achieved using the following gradient: 0 min, 5%B; 2 min, 5%B; 4 min, 25% B; 8 min, 50% B; 10 min, 80% B; 14 min, 100% B; 15 min,100% B; 15.1 min, 5% and 16 min, 5%B. The flow rate was 0.35 mL/min, and the column temperature was 45 °C. All the samples were kept at 4 °C during the analysis. The injection volume was 10 μL. The mass range was from *m*/*z* 125 to 1000. The resolution was set at 70,000 for the full MS scans and 17,500 for HCD MS/MS scans. The collision energy was set at 10, 20, and 40 eV. The mass spectrometer operated as follows: spray voltage, 3500 V (+) and 3500 V (−); sheath gas flowrate, 40 arbitrary units (+) and 35 arbitrary units (−); auxiliary flowrate, 10 arbitrary units (+) and 8 arbitrary units (−); capillary temperature, 320 °C. The QCs were injected every 6 samples throughout the analytical run to provide a set of data from which repeatability can be assessed.

### 3.10. Data Processing and Statistical Analysis

The data reported in this work were the average of three independent experiments, which are expressed as means ± standard deviations. To detect whether there are significant differences in the contents of antioxidant components and bioactivities, a one-way analysis of variance using the procedures of Duncan’s multiple comparisons test of IBM SPSS Statistics (SPSS Vers. 26.0; SPSS Inc., Chicago, IL, USA), similar to our recent work [39]. HPLC fingerprinting was established by importing the chromatographic data of 16 batches of samples into the Similarity Evaluation System for Chromatographic Fingerprint of Traditional Chinese Medicine software (Vers. 2012), identifying common characteristic peaks which were those detected in all the fingerprints of the samples. Based on the simulative median chromatogram. The similarity index was also calculated to assess. The models for unsupervised principal component analysis (PCA) and PLSR were established by SIMCA-P + version 14.1 (UMETRICS AB, Ume, Vasterbotten, Sweden).

## 4. Conclusions

This investigation compared the chromatographic fingerprints and bioactivities of antioxidants and enzyme-inhibitory activities between *Flos Chrysanthemum* (JH) and wild *Chrysanthemum* (YJH), followed by the establishment of fingerprint–efficacy relationship models. Both types of *Chrysanthemums* belong to the *Asteraceae* family and share some similarities in appearance and medicinal properties, which are both used as traditional Chinese medicine in many TCM formulas, although they may have different growth habits and colors. However, the compositional differences have been scarcely characterized, not to mention their fingerprint–efficacy relationship. This work concluded that relying on chromatographic fingerprints achieved the complete separation of different types of *Chrysanthemum* samples. The concentrations of total phenolics and flavonoids of JH samples were shown to be generally higher than those of YJH, but the concentration distribution ranges of YJH were significantly greater compared to JH, possibly related to environmental stress factors leading to the concentration fluctuations of phytochemicals during the growth and flowering of *Chrysanthemum* cultivars. Correspondingly, the total antioxidant capabilities of JH were greatly higher than those of YJH samples, as revealed by chemical assays, including DPPH and ABTS radical scavenging activities and FRAP assays. In addition, cellular-based antioxidant activities confirmed the results of chemical assays, suggesting that the differences in antioxidant activities among the different types of *Chrysanthemums* were obvious. The extracts from YJH and JH samples showed significant α-glucosidase inhibitory activity and lipase-inhibitory activity, implying the modulatory effects on lipid and glucose metabolisms, which were also confirmed by an untargeted cell-based metabolomics approach. The selected common peaks by similarity analysis contributed to the discrimination of YJH and JH samples, and the modeling of the fingerprint–bioactivity relationship identified neochlorogenic acid, isochlorogenic acid A, and linarin as efficacy-associated chemical markers. It should be noted that although the fingerprint–efficacy relationship was achieved and efficient for quality consistency evaluation and efficacy prediction, the reproducibility of specific pharmacological attributes requires further verification in future research.

## Data Availability

Data will be made available on request.

## Supplementary Materials

The following supporting information can be downloaded at: https://www.mdpi.com/article/10.3390/molecules28176254/s1, Table S1: Information about the sources of *Flos Chrysanthemum* and wild *Chrysanthemum* (*Chrysanthemum indicum* L.).

## References

[1] Wang F., Xiong Z.-Y., Li P., Yang H., Gao W., Li H.-J. (2017). From chemical consistency to effective consistency in precise quality discrimination of Sophora flower-bud and Sophora flower: Discovering efficacy-associated markers by fingerprint-activity re-lationship modeling. J. Pharm. Biomed. Anal..

[2] Gu J., Scotti F., Reich E., Kirchhof R., Booker A., Heinrich M. (2022). Chrysanthemum species used as food and medicine: Understanding quality differences on the global market. S. Afr. J. Bot..

[3] Liang F., Hu C., He Z., Pan Y. (2014). An arabinogalactan from flowers of *Chrysanthemum morifolium*: Structural and bioactivity studies. Carbohydr. Res..

[4] Hodaei M., Rahimmalek M., Arzani A., Talebi M. (2018). The effect of water stress on phytochemical accumulation, bioactive compounds and expression of key genes involved in flavonoid biosynthesis in *Chrysanthemum morifolium* L.. Ind. Crops Prod..

[5] Lin L.-Z., Harnly J.M. (2010). Identification of the phenolic components of chrysanthemum flower (*Chrysanthemum morifolium* Ramat). Food Chem..

[6] Yuan H., Luo J., Lyu M., Jiang S., Qiu Y., Tian X., Liu L., Liu S., Ouyang Y., Wang W. (2022). An integrated approach to Q-marker discovery and quality assessment of edible *Chrysanthemum* flowers based on chromatogram–effect relationship and bioin-formatics analyses. Ind. Crops Prod..

[7] Chu Q., Fu L., Guan Y., Ye J. (2004). Determination and Differentiation of *Flos Chrysanthemum* Based on Characteristic Elec-trochemical Profiles by Capillary Electrophoresis with Electrochemical Detection. J. Agric. Food Chem..

[8] Maitreesophone P., Khine H.E.E., Nealiga J.Q.L., Kongkatitham V., Panuthai P., Chaotham C., Likhitwitayawuid K., Sritularak B. (2022). α-Glucosidase and pancreatic lipase inhibitory effects and anti-adipogenic activity of dendrofalconerol B, a bisbibenzyl from Dendrobium harveyanum. S. Afr. J. Bot..

[9] Yang C., Zheng Y., Green B.D., Zhou C., Pan D., Cao J., Wang L., Cai Z., Xia Q. (2022). Volatilome evolution during storage and in vitro starch digestibility of high-power ultrasonication pretreated wholegrain *Oryza sativa* L.. Food Res. Int..

[10] Papoutsis K., Zhang J., Bowyer M.C., Brunton N., Gibney E.R., Lyng J. (2021). Fruit, vegetables, and mushrooms for the preparation of extracts with α-amylase and α-glucosidase inhibition properties: A review. Food Chem..

[11] Elbashir S.M.I., Devkota H.P., Wada M., Kishimoto N., Moriuchi M., Shuto T., Misumi S., Kai H., Watanabe T. (2018). Free radical scavenging, α-glucosidase inhibitory and lipase inhibitory activities of eighteen Sudanese medicinal plants. BMC Complement. Altern. Med..

[12] Zhang C., Zheng X., Ni H., Li P., Li H.-J. (2018). Discovery of quality control markers from traditional Chinese medicines by fingerprint-efficacy modeling: Current status and future perspectives. J. Pharm. Biomed. Anal..

[13] Zhang J., Wang J., Yang L., Wang Y., Jin W., Li J., Zhang Z. (2023). Comprehensive Quality Evaluation of Polygonatum cyrtonema and Its Processed Product: Chemical Fingerprinting, Determination and Bioactivity. Molecules.

[14] Yang L., Xie X., Yang L., Zhang J., Sun G. (2016). Monitoring quality consistency of Ixeris sonchifolia (Bunge) Hance injection by integrating UV spectroscopic fingerprints, a multi-wavelength fusion fingerprint method, antioxidant activities and UHPLC/Q-TOF-MS. RSC Adv..

[15] Yuan J.-h., Cai Z.-C., Chen C.-H., Wu N., Yin S.-X., Wang W.-X., Chen H.-J., Zhou Y.-Y., Li L., Liu X.-H. (2022). A study for quality evaluation of Taxilli Herba from different hosts based on fingerprint-activity relationship modeling and multivariate statistical analysis. Arab. J. Chem..

[16] Tian D., Yang Y., Yu M., Han Z.-Z., Wei M., Zhang H.-W., Jia H.-M., Zou Z.-M. (2020). Anti-inflammatory chemical constituents of *Flos Chrysanthemi Indici* determined by UPLC-MS/MS integrated with network pharmacology. Food Funct..

[17] Mahajan M., Kuiry R., Pal P.K. (2020). Understanding the consequence of environmental stress for accumulation of secondary metabolites in medicinal and aromatic plants. J. Appl. Res. Med. Aromat. Plants.

[18] Shao Y., Sun Y., Li D., Chen Y. (2020). *Chrysanthemum indicum* L.: A comprehensive review of its botany, phytochemistry and pharmacology. Am. J. Chin. Med..

[19] Xia Q., Zheng Y., Wang L., Chen X. (2023). Proposing signaling molecules as key optimization targets for intensifying the phy-tochemical biosynthesis induced by emerging nonthermal stress pretreatments of plant-based foods: A focus on gam-ma-aminobutyric acid. J. Agric. Food Chem..

[20] Martinelli E., Granato D., Azevedo L., Gonçalves J.E., Lorenzo J.M., Munekata P.E., Simal-Gandara J., Barba F.J., Carrillo C., Rajoka M.S.R. (2021). Current perspectives in cell-based approaches towards the definition of the antioxidant activity in food. Trends Food Sci. Technol..

[21] Wang H., Guo X., Hu X., Li T., Fu X., Liu R.H. (2017). Comparison of phytochemical profiles, antioxidant and cellular antioxidant activities of different varieties of blueberry (*Vaccinium* spp.). Food Chem..

[22] Wang A., Hou K., Mu G., Ma C., Tuo Y. (2021). Antioxidative effect of soybean milk fermented by Lactobacillus plantarum Y16 on 2,2–azobis (2-methylpropionamidine) dihydrochloride (ABAP)-damaged HepG2 cells. Food Biosci..

[23] Xia Q., Green B.D., Zhu Z., Li Y., Gharibzahedi S.M.T., Roohinejad S., Barba F.J. (2019). Innovative processing techniques for altering the physicochemical properties of wholegrain brown rice (*Oryza sativa* L.)—Opportunities for enhancing food quality and health attributes. Crit. Rev. Food Sci. Nutr..

[24] Tan Y., Chang S.K.C., Zhang Y. (2017). Comparison of α-amylase, α-glucosidase and lipase inhibitory activity of the phenolic substances in two black legumes of different genera. Food Chem..

[25] Shawul A.A., Chakma S., Melesse A.M. (2019). The response of water balance components to land cover change based on hy-drologic modeling and partial least squares regression (PLSR) analysis in the Upper Awash Basin. J. Hydrol. Reg. Stud..

[26] Jia J., Deng H., Duan J., Zhao J. (2009). Analysis of the major drivers of the ecological footprint using the STIRPAT model and the PLS method—A case study in Henan Province, China. Ecol. Econ..

[27] Ruan Y., Cai Z., Deng Y., Pan D., Zhou C., Cao J., Chen X., Xia Q. (2021). An untargeted metabolomic insight into the high-pressure stress effect on the germination of wholegrain *Oryza sativa* L.. Food Res. Int..

[28] Xia Q., Wang L., Xu C., Mei J., Li Y. (2017). Effects of germination and high hydrostatic pressure processing on mineral elements, amino acids and antioxidants in vitro bioaccessibility, as well as starch digestibility in brown rice (*Oryza sativa* L.). Food Chem..

[29] Han C., Zheng Y., Wang L., Zhou C., Wang J., He J., Sun Y., Cao J., Pan D., Xia Q. (2023). Contribution of process-induced molten-globule state formation in duck liver protein to the enhanced binding ability of (E,E)-2,4-heptadienal. J. Sci. Food Agric..

[30] Xia Q., Zheng Y., Liu Z., Cao J., Chen X., Liu L., Yu H., Barba F.J., Pan D. (2020). Nonthermally driven volatilome evolution of food matrices: The case of high pressure processing. Trends Food Sci. Technol..

[31] Xia Q., Mei J., Yu W., Li Y. (2017). High hydrostatic pressure treatments enhance volatile components of pre-germinated brown rice revealed by aromatic fingerprinting based on HS-SPME/GC–MS and chemometric methods. Food Res. Int..

[32] Worley B., Powers R. (2016). PCA as a Practical Indicator of OPLS-DA Model Reliability. Curr. Metabolomics.

[33] Liu M., Gong X., Quan Y., Zhou Y., Li Y., Peng C. (2019). A Cell-Based Metabonomics Approach to Investigate the Varied Influences of Chrysophanol-8-O-β-D-Glucoside with Different Concentrations on L-02 Cells. Front. Pharmacol..

[34] García-Cañaveras J.C., Jiménez N., Gómez-Lechón M.J., Castell J.V., Donato M.T., Lahoz A. (2015). LC-MS untargeted metab-olomic analysis of drug-induced hepatotoxicity in HepG2 cells. Electrophoresis.

[35] Wolfe K.L., Liu R.H. (2007). Cellular Antioxidant Activity (CAA) Assay for Assessing Antioxidants, Foods, and Dietary Supplements. J. Agric. Food Chem..

[36] Dion M.Z., Wang Y.J., Bregante D., Chan W., Andersen N., Hilderbrand A., Leiske D., Salisbury C.M. (2018). The Use of a 2,2′-Azobis (2-Amidinopropane) Dihydrochloride Stress Model as an Indicator of Oxidation Susceptibility for Monoclonal Antibodies. J. Pharm. Sci..

[37] Uraipong C., Zhao J. (2016). Rice bran protein hydrolysates exhibit strong in vitro α-amylase, β-glucosidase and ACE-inhibition activities. J. Sci. Food Agric..

[38] Maqsood M., Ahmed D., Atique I., Malik W. (2017). Lipase inhibitory activity of Lagenaria siceraria fruit as a strategy to treat obesity. Asian Pac. J. Trop. Med..

[39] Han C., Zheng Y., Zhou C., Liang Y., Huang S., Sun Y., Cao J., He J., Barba F.J., Liu J. (2023). Pressurization-induced molten globule-like protein as a regulatory target for off-flavor controlling: Thermodynamic evidence and molecular modeling. Food Control.

