# Supplementary material for: Identification of Efficacy-Associated Markers to Discriminate Flos Chrysanthemum and Flos Chrysanthemi Indici Based on Fingerprint–Activity Relationship Modeling: A Combined Evaluation over Chemical Consistence and Quality Consistence
Source: Molecules. 2023 Aug 25;28(17):6254. doi: 10.3390/molecules28176254 (PMC10488643; doi:10.3390/molecules28176254)
Supplement: Supplementary file 1 [file molecules-28-06254-s001.zip › molecules-2553870-supplementary.pdf]

Supplementary information

Identification of Efficacy-Associated Markers to Discriminate *Flos Chrysanthemum* and *Flos Chrysanthemi Indici* Based on Fingerprint–Activity Relationship Modeling: A Combined Evaluation over Chemical Consistence and Quality Consistence

Feng Liu [1], Yuanrong Zheng [2], Huijie Hong [3], Lianliang Liu [4], Xiaojia Chen [3,*] and Qiang Xia [3,4,*]

1 Department of Horticultural Technology, Ningbo City College of Vocational Technology,
Ningbo 315100, China
2 State Key Laboratory of Dairy Biotechnology, Shanghai Engineering Research Center of Dairy Biotechnology, Dairy Research Institute, Bright Dairy & Food Co., Ltd., Shanghai 200436, China
3 State Key Laboratory of Quality Research in Chinese Medicine, Institute of Chinese Medical Sciences,
University of Macau, Macau 999078, China
4 College of Food and Pharmaceutical Sciences, Key Laboratory of Animal Protein Food Processing
Technology of Zhejiang Province, Ningbo University, Ningbo 315832, China
* Correspondence: xiaojiachen@um.edu.mo (X.C.); xiaqiang@nbu.edu.cn or xiaqiangnn@outlook.com (Q.X.)

Table S1. Information about the sources of *Flos Chrysanthemum* and wild *Chrysanthemum* (*Chrysanthemum indicum* L.).

Sample no.	Variety	Geographical location	Sources
S1	*Wild Chrysanthemum*	Hubei	Zhonggangda pharmacy
S2	*Wild Chrysanthemum*	Shangluo, Shangxi	Lijinghua
S3	*Wild Chrysanthemum*	Henan	Suxianshi
S4	*Wild Chrysanthemum*	Tibet	Zangxitang
S5	*Wild Chrysanthemum*	Sichuan	Xunbaicao
S6	*Wild Chrysanthemum*	Anhui	Songbaicao
S7	*Wild Chrysanthemum*	Guilin, Guangxi	Retail pharmacy
S8	*Wild Chrysanthemum*	Zhejiang	Guicheng
S9	*Wild Chrysanthemum*	Anhui	Shichuntang
S10	*Flos Chrysanthemum (Chuju)*	Chuzhou, Anhui	Retail pharmacy
S11	*Flos Chrysanthemum (Huai*	Jiaozhu, Henan	Retail pharmacy

	Chrysanthemum)		
S12	*Flos Chrysanthemum*	Tongxiang, Zhejiang	Retail pharmacy
S13	*Flos Chrysanthemum (Tribute chrysanthemum)*	Huangshan, Anhui	Retail pharmacy
S14	*Flos Chrysanthemum*	Bo Zhou, Anhui	Retail pharmacy
S15	*Flos Chrysanthemum*	Jiangsu	Retail pharmacy
S16	*Flos Chrysanthemum*	Zhejiang	Dongziyunxuan
